# Deciphering key features in protein structures with the new ENDscript server

**DOI:** 10.1093/nar/gku316

**Published:** 2014-04-21

**Authors:** Xavier Robert, Patrice Gouet

**Affiliations:** Equipe de Biocristallographie et Biologie Structurale des Cibles Thérapeutiques, IBCP-BMSSI, UMR 5086 CNRS Université de Lyon, SFR BioSciences Gerland-Lyon Sud, 7 Passage du Vercors, 69367 Lyon Cedex 07, France

## Abstract

ENDscript 2 is a friendly Web server for extracting and rendering a comprehensive analysis of primary to quaternary protein structure information in an automated way. This major upgrade has been fully re-engineered to enhance speed, accuracy and usability with interactive 3D visualization. It takes advantage of the new version 3 of ESPript, our well-known sequence alignment renderer, improved to handle a large number of data with reduced computation time. From a single PDB entry or file, ENDscript produces high quality figures displaying multiple sequence alignment of proteins homologous to the query, colored according to residue conservation. Furthermore, the experimental secondary structure elements and a detailed set of relevant biophysical and structural data are depicted. All this information and more are now mapped on interactive 3D PyMOL representations. Thanks to its adaptive and rigorous algorithm, beginner to expert users can modify settings to fine-tune ENDscript to their needs. ENDscript has also been upgraded as an open platform for the visualization of multiple biochemical and structural data coming from external biotool Web servers, with both 2D and 3D representations. ENDscript 2 and ESPript 3 are freely available at http://endscript.ibcp.fr and http://espript.ibcp.fr, respectively.

## INTRODUCTION

Sequence similarity search and multiple sequence alignment (MSA) are methods of choice for solving or interpreting bioinformatics and biological problems (1). They can address many questions and thus are the basis of numerous evolutionary and comparative studies, e.g. homology identification, protein function, structure and interaction predictions, computer-aided mutagenesis, phylogenetic analyses, etc. Over the years, the exponential growth of sequence and structure databases content (2) has raised several challenging issues concerning, e.g. data curation, storage, search, accessibility and visualization.

The Web server ENDscript 1 (3,4) was created in 2002 to facilitate the generation of figures containing a large amount of protein sequences and secondary structure information. Since then, similar programs have been released with extended functionalities (e.g. STRAP (5), Aline (6), Jalview 2 (7) or PROMALS3D (8)). ENDscript is derived from our well-known ESPript program that renders sequence similarities and secondary structure elements from aligned sequences with numerous options to optimize and enhance their depiction. ESPript version 1 (9) and 2 (4) belong to a family of programs developed for non-interactive MSAs representation (e.g. Alscript (10), BoxShade or PRALINE (11)). To date, several biotool Web servers are interfaced with ESPript: connections are available from T-Coffee (12), NPS@ (13), MultAlin (14) (each performing sequence analyses and similarity searches) and from the protein domain database ProDom (15).

Here, we present ENDscript 2, a friendly Web server for extracting and rendering a comprehensive analysis of primary to quaternary protein structure information in an automated way. The new ENDscript 2 server (http://endscript.ibcp.fr) is a major upgrade of ENDscript 1.x, which has been entirely re-engineered to be faster and to process information from thousand of sequences and structures thanks to a parallel programming. It can now generate, with a few mouse clicks and without queuing, high quality figures with the new version 3 of ESPript (http://espript.ibcp.fr) as well as interactive 3D representations with a novel automated PyMOL script generator. 

## INPUT AND OUTPUTS

ENDscript uses as query either a four-digit Protein Data Bank (PDB) identifier (16) or an uploaded coordinate file conforming to the PDB format (nuclear magnetic resonance (NMR), crystallographic structures and protein models are supported). From this query, ENDscript generates four distinct downloadable illustrations.

**Figure 1.**
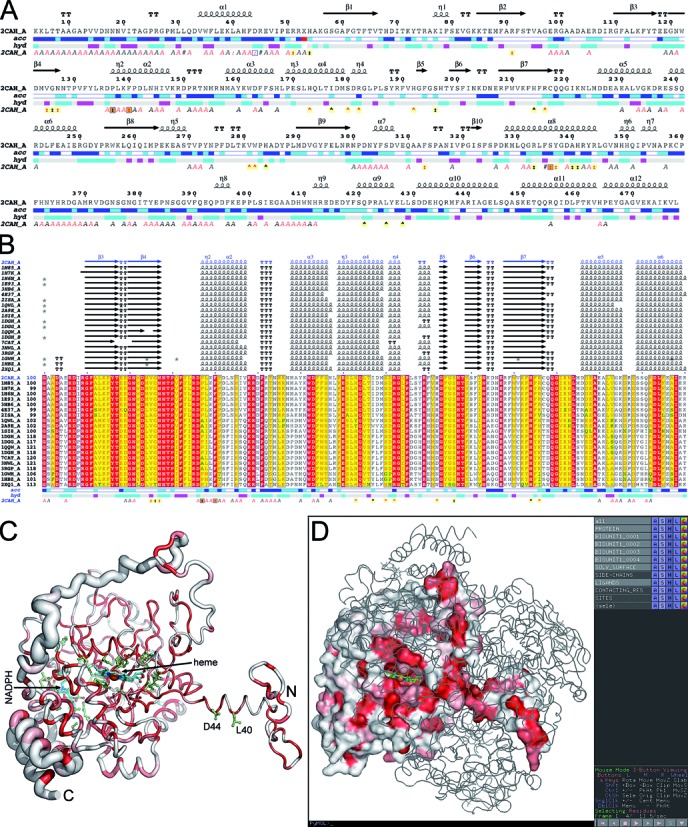
Sample of ENDscript representations obtained from the PDB entry 2CAH. (**A**) Flat figure showing the sequence of 2CAH with secondary structure elements presented on top (helices with squiggles, β-strands with arrows and turns with TT letters). Solvent accessibility is rendered by a first bar below the sequence (blue is accessible, cyan is intermediate, white is buried) and hydropathy by a second bar below (pink is hydrophobic, white is neutral, cyan is hydrophilic). Bottom letters and symbols depict crystallographic, protein:protein and protein:ligand contacts (see ‘Case study’). (**B**) Excerpt from the second ENDscript flat figure, showing the MSA of the 20 most homologous proteins to 2CAH (obtained with a BLAST+ search against the PDBAA database). Known secondary structure elements are displayed for all aligned sequences. Alternate residues are highlighted by gray stars. Identical and similar residues are boxed in red and yellow, respectively. (**C**) The ‘Sausage’ PyMOL representation showing a tube depiction of 2CAH, whose radius is proportional to the mean rms deviation per residue between Cα pairs. The tube is colored according to the level of sequence conservation, from white (low score) to red (identity). The heme cofactor and bound NADPH are presented in cyan ball-and-stick. Their contacting residues are presented in light green ball-and-stick. (**D**) Screen capture of a PyMOL session showing the biological tetrameric assembly of 2CAH. One monomer is presented by its solvent accessible surface with the same color ramping and orientation as in panel (C). The Cα traces of the three other monomers are presented. On the right, the PyMOL control panel with preset buttons allows to show and hide structural features compiled by ENDscript.

The first one is a flat figure presenting the amino acid sequence of the PDB query adorned with secondary structure elements, solvent accessibility and hydropathy scales per residue (see an example in Figure 1A). In addition and if applicable, non-crystallographic and crystallographic protein:ligand and protein:protein contacts as well as disulfide bridges are highlighted by specific markers. Several common hetero-compounds are automatically kept and are subsequently depicted by given symbols on the figure. The user can manually keep non-recognized hetero-compounds or monatomic elements contained in his PDB query.

The second illustration is also a flat figure showing, in addition to the previous information, an MSA of proteins homologous to the query and colored according to residue conservation (see an excerpt in Figure 1B). The experimental secondary structure elements of each identified homologous protein are depicted at the top of the sequence block if their 3D structures are known.

The third and fourth illustrations consist in two interactive 3D representations of the PDB query. To take advantage of these latter, the program PyMOL (The PyMOL Molecular Graphics System, Schrödinger LLC, http://www.pymol.org) has to be installed on the user's computer.

The first PyMOL representation is named ‘Cartoon’: this is a ribbon depiction of the PDB query colored as a function of similarity scores calculated from the preceding MSA. The color ramping from white (low score) to red (identity) allows to quickly locate areas of weak and strong sequence conservation on the query structure. The second PyMOL representation is titled ‘Sausage’: it shows a variable tube depiction of the Cα trace of the query (see an example in Figure 1C). For this representation, all homologous protein structures were superimposed on the PDB query and the radius of the tube is proportional to the mean rms deviation per residue between Cα pairs. The same white to red color ramping is used to visualize possible substitutions in sequence. Hence, the user can identify areas of weak and strong structural conservation and correlate this result with sequence conservation.

For these two PyMOL representations, a solvent-accessible surface mapped with the same coloring code can be switched on or off via the PyMOL control panel (see an example in Figure 1D). If applicable, the user can also display an assortment of supplementary information compiled by ENDscript: biological assembly and multiple NMR models; disulfide bridges; nucleic acids, ligands or monatomic elements and their contacting residues; strictly conserved residues; PDB SITES markers.

All these features are editable thanks to the PyMOL graphical user interface, and publication-quality pictures can rapidly be obtained with its built-in ray-tracing function. Depending on his skill in PyMOL usage, the user has the choice to download an all-in-one and ready-to-use PyMOL .pse session file, or a .zip archive file containing a PyMOL .pml script and associated required files to manually edit them to his need. Therefore, the user has full flexibility to customize these representations automatically produced by ENDscript.

In addition, an MSA file with secondary structure elements in Stockholm format is generated by ENDscript and can be examined (and modified) online with the JalviewLite viewer (7). Finally, hypertext links to the PDB record of each identified protein of known structure similar to the query are presented. The user can download a .zip file containing all these latter individually superimposed on the PDB query.

## NEW FEATURES AND IMPROVEMENTS

### Changes and new features in ESPript program and Web server

The ENDscript Web server uses the core Fortran program ESPript in its backend. The new version 3 of ESPript has been deeply modified to fulfill two key requirements driven by ENDscript 2: minimizing its execution time and handling a large number of data.

For this purpose, OpenMP parallelization directives have been added to the PostScript rendering core routines of ESPript in order to take advantage of multicore and multiprocessor architectures. Hence, the resulting MSA PostScript files are no more rendered line-by-line but in parallel by using as many cores/processors the machine has. As a result, the execution time bottleneck constituted by the rendering process has been drastically reduced, this permitting the rapid depiction without queuing of up to 2500 aligned sequences decorated with their secondary structure elements on 4500 columns.

In addition, ESPript 3 now runs on an x86_64 platform, allowing the program to address memory in a more efficient way and to handle very large tables. The program was compiled with gfortran, part of the GNU Compiler Collection, with the *-mcmodel = medium* flag to allocate tables exceeding 2GB. This choice was essential as the data that ESPript is led to manage is growing proportionally to the number of sequences and structures in databases implemented in ENDscript.

The ESPript graphical interface has been considerably revamped in order to better meet the needs of the users who are now guided through the whole process by tooltips, detailed help topics and a new tutorial accessible at any time. New coloring schemes and markers have been implemented as well as output and paper size formats: ESPript renders publication-quality illustrations in most common file formats (PostScript, PDF, PNG, TIFF) and sizes (US letter, A4, A3, A0 and the ‘Tapestry’ format—0.8 × 3.3 m—ideally fitted for depicting huge MSAs).

### New features and enhancements in ENDscript Web server

Even if the general process scheme remains unchanged between version 1 and 2 of ENDscript, we considerably re-engineered the initial code to enhance speed, accuracy and usability.

In this goal, the new ESPript 3 program, described above, was of primary importance. In addition, helper programs, part of the ENDscript pipeline, were chosen for their efficiency and their ability to use multicore machines. Hence, we replaced the programs BLAST and ClustalW2 by their latest revisions (BLAST+ (17) and Clustal Omega (18), respectively) to gain in scalability, accuracy and performance. In the same goal, two MSA programs were added to the ENDscript pipeline, namely MAFFT (19) and MSAProbs (20).

In the previous version of ENDscript, only two sequence databases were available (UniProtKB/Swiss-Prot and PDBAA, the sequence database derived from the PDB). In ENDscript 2, we added 12 complete genome databases, the UniProtKB/TrEMBL as well as the PDBAA95, PDBAA90, PDBAA70 and PDBAA50 containing the results of the clustering of protein chains in the PDB at 95%, 90%, 70% and 50% sequence identity, respectively (see Supplementary Table S1).

As for the ESPript Web server, the ENDscript graphical user interface was utterly remodeled for improved usability with tooltips, detailed help topics and tutorial. The results, separated in hierarchical sections, are now presented in a clearer manner. Finally, we opted for the molecular visualization program PyMOL as default viewer for the interactive 3D representations as it is multi-platform and widely adopted by biologists and structural biologists. ENDscript takes full advantage of the convenient PyMOL control panel, notably of its ability to show and hide information or objects with a single click, this facilitating the analysis of the results. In consequence, the *PyMOL-ScriptMaker* subroutine now supersedes the previous MolScript/Bobscript scripting feature of ENDscript 1.

### ENDscript is open to external bioinformatics services

In addition to the above-mentioned enhancements and new features, we designed ENDscript 2 as an open platform for the visualization of multiple biochemical and structural information. Indeed, ENDscript has the ability, for its primary to quaternary structure representations, to depict a large range of supplementary data coming from external sources. Hence, any biotool Web server calculating or predicting structure-related properties can use ENDscript for an effective display. This implies that ENDscript is supplied with a modified PDB file conforming to some simple annotation conventions and modifications. In practice, the property to be displayed by ENDscript must be numerically expressed per residue or per atom in order to replace the B-factor or occupancy values. A REMARK record in the PDB file header must specify a keyword associated to the interfaced service as well as the minimal and maximal values of the property—this allowing ENDscript to normalize the data. In concrete terms, colored bars or special characters in the flat representations can present this supplementary information. It can also be displayed in 3D with PyMOL by highlighting the appropriate residues or atoms with specific coloring scheme and/or by any other means (spheres, dots, meshes, surfaces, etc.).

A proof of concept of this new ENDscript feature is presented in Supplementary Data 1. Biotool Web server administrators interested in interfacing their service with ENDscript can contact the authors to obtain the annotation rules and the connection protocol.

## CASE STUDY

Catalases are tetrameric heminic enzymes that decompose hydrogen peroxide into water and oxygen. They serve to protect cells from the toxic effects of hydrogen peroxide. As a case study, we used ENDscript to decipher features in the crystal structure of *Proteus mirabilis* catalase (PDB entry 2CAH) (21).

The two fundamental residues of the heme-iron catalytic site are a distal histidine and a proximal tyrosine. A unique methionine sulfone is observed in the distal site of *P. mirabilis* catalase. Another peculiarity of this bacterial catalase is its ability to bind nicotinamide adenine dinucleotide phosphate (NADPH) for the prevention of inactivation by hydrogen peroxide (22).

The first flat figure clearly shows that the catalase structure has an α+β topology (Figure 1A and Supplementary Data 2). Moreover, the N-terminal region is involved in extensive protein:protein crystallographic contacts as shown by italic A letters below the sequence block. A red letter identifies a contact < 3.2 Å while a black letter identifies a contact between 3.2 and 5 Å. Leu31 is positioned along a 2-fold crystallographic axis as shown by an italic hash symbol. Leu40 and Asp44 are in contact with the heme group of a symmetric monomer as shown by an italic colon. Asp44 is also involved in a protein:protein crystallographic contact as shown by a blue frame. Arg51 binds the heme group of the monomer as shown by a normal colon on a light yellow background. The non-standard residue in position 53 (labeled X) is the methionine sulfone of the distal site. Phe140 may be a critical residue: it has close contacts with the heme group as shown by the red colon. It is also involved in protein:protein contacts as shown by the blue frame. Finally, it is involved both in crystallographic and non-crystallographic contacts as shown by the orange background. His173 binds tightly NADPH as shown by a red caret. The second flat figure (Figure 1B and Supplementary Data 3) reveals that secondary structure elements are well conserved in catalases deposited in the PDB.

The 3D ‘Sausage’ representation (Figure 1C and Supplementary File 1) allows for emphasizing the Cα trace of the C-terminal region that varies between catalases (the tube radius is proportional to the mean rms deviation between 2CAH and homologous catalases). In agreement, the C-terminal region is poorly conserved in sequence and is mainly colored in white (color ramping from white, low conservation, to red, identity). By contrast the N-terminal region, which protrudes out of the protein core, is surprisingly well conserved. In fact, this region is deeply buried in the biological tetramer, this being visible by displaying the biological assembly (Figure 1D and Supplementary File 1) automatically incorporated by ENDscript in the PyMOL session file. Finally, thanks to a preset button on its control panel, PyMOL can display the protein solvent accessible surface colored according to the level of sequence conservation (Figure 1D).

## CONCLUSION

ENDscript 2 is a major update of our joint ESPript/ENDscript Web service, which has benefited over the past decade of a mean of 1500 unique visitors a month from more than 25 countries submitting over 14 000 jobs monthly. It takes advantage of the new version 3 of ESPript, which previous releases total more than 2000 combined cross-citations in ‘Web of Science’ and are supported by the SBGrid Consortium (Harvard Medical School, http://www.sbgrid.org).

The new ENDscript Web server has been conceived to be fast and convenient. Thanks to its automated pipeline and a parallel programming, ENDscript can deliver most results within 1 min. No particular knowledge in bioinformatics is needed to obtain comprehensive and relevant illustrations. However, demanding or expert users can modify settings to fine-tune ENDscript to their needs.

For all these reasons, ENDscript is a tool of choice for biologists and structural biologists, which allows for generating with a few mouse clicks a set of detailed high quality figures and interactive 3D representations of their proteins of interest. With its simplified interface and its ability to quickly render the key features of any protein structure, ENDscript is also an effective educational tool, which can be used during courses or practical work sessions in structural biology.

## SUPPLEMENTARY DATA

Supplementary Data are available at NAR Online.

Supplementary Data
