# Supplementary material for: Deciphering key features in protein structures with the new ENDscript server
Source: Nucleic Acids Res. 2014 Apr 21;42(Web Server issue):W320–4. doi: 10.1093/nar/gku316 (PMC4086106; doi:10.1093/nar/gku316)
Supplement: Supplementary Data [file supp_42_W1_W320__index.html]

Supplementary Data 

# Deciphering key features in protein structures with the new ENDscript server

## Supplementary Data

**Files in this Data Supplement:**

- SUPPLEMENTARY DATA
- SUPPLEMENTARY DATA
- SUPPLEMENTARY DATA
- SUPPLEMENTARY DATA
- SUPPLEMENTARY DATA
