# Supplementary material for: Deciphering key features in protein structures with the new ENDscript server
Source: Nucleic Acids Res. 2014 Apr 21;42(Web Server issue):W320–4. doi: 10.1093/nar/gku316 (PMC4086106; doi:10.1093/nar/gku316)
Supplement: Supplementary Data [file supp_gku316_nar-00222-web-b-2014-File003.pdf]

Supplementary Table 1.

List of sequence and protein structure databases implemented in the ENDscript server. Sequence databases are used by ENDscript to detect proteins homologous to the PDB query (PDBAA is the default). Protein structure and internal databases are employed to speed-up the ENDscript process and to gather additional information compiled in the resulting data and files. All these databases are automatically updated on a weekly basis.

**Database name**	**Description**	**Source**
	Sequence databases	
ARATH	Complete proteome from *Arabidopsis thaliana*	UniProtKB/Swiss-Prot
BOVIN	Complete proteome from *Bos taurus*	UniProtKB/Swiss-Prot
CAEEL	Complete proteome from *Caenorhabditis elegans*	UniProtKB/Swiss-Prot
CANFA	Complete proteome from *Canis familiaris*	UniProtKB/Swiss-Prot
CHICK	Complete proteome from *Gallus gallus*	UniProtKB/Swiss-Prot
DANRE	Complete proteome from *Danio rerio*	UniProtKB/Swiss-Prot
DROME	Complete proteome from *Drosophila melanogaster*	UniProtKB/Swiss-Prot
HUMAN	Complete proteome from *Homo sapiens*	UniProtKB/Swiss-Prot
MOUSE	Complete proteome from *Mus musculus*	UniProtKB/Swiss-Prot
PDBAA	Sequences derived from PDB protein structures	NCBI/RCSB PDB
PDBAA50	Clustering of PDB protein chains at 50% sequence identity	Maintained by the ENDscript server
PDBAA70	Clustering of PDB protein chains at 70% sequence identity	Maintained by the ENDscript server
PDBAA90	Clustering of PDB protein chains at 90% sequence identity	Maintained by the ENDscript server
PDBAA95	Clustering of PDB protein chains at 95% sequence identity	Maintained by the ENDscript server
PIG	Complete proteome from *Sus scrofa*	UniProtKB/Swiss-Prot
RAT	Complete proteome from *Rattus norvegicus*	UniProtKB/Swiss-Prot
SWISSPROT	Non-redundant, manually annotated and reviewed	UniProtKB/Swiss-Prot
TREMBL	Automatically annotated and not reviewed	UniProtKB/TrEMBL
YEAST	Complete proteome from *Saccharomyces cerevisiae*	UniProtKB/Swiss-Prot
	Protein structure databases	
PDB	PDB coordinate files	RCSB PDB
PDB/BIOUNIT	PDB biological assembly coordinate files	RCSB PDB
	Internal databases	
END_SPDB_DB	PDB coordinate files processed by the program SPDB	Maintained by the ENDscript server
END_DSSP_DB	PDB coordinate files processed by the program DSSP	Maintained by the ENDscript server

23. Martin, J. and Lavery, R. (2012) Arbitrary protein-protein docking targets biologically relevant interfaces. BMC biophysics, 5, 7.
24. Ritchie, D.W. and Venkatraman, V. (2010) Ultra-fast FFT protein docking on graphics processors. Bioinformatics (Oxford, England), 26, 2398-2405.
