# Supplementary material for: Deciphering key features in protein structures with the new ENDscript server
Source: Nucleic Acids Res. 2014 Apr 21;42(Web Server issue):W320–4. doi: 10.1093/nar/gku316 (PMC4086106; doi:10.1093/nar/gku316)
Supplement: Supplementary Data [file supp_gku316_nar-00222-web-b-2014-File004.pdf]

Supplementary Data 1.

ENDscript is open to external bioinformatics services - Proof of concept:

It has been demonstrated that docking target proteins against an arbitrary set of proteins leads to a non-random localization of interaction interfaces (23). In consequence, an approach using arbitrary docking, and based solely on physical properties, can successfully identify biologically pertinent protein interfaces. In collaboration with Martin & Lavery (23), this approach was used on the crystal structure of *Proteus mirabilis* catalase as a test case (PDB entry 2CAH). This latter structure was submitted to a protein:protein docking experiment using the Hex software (24) against a set of 25 small proteins. The resulting docking poses were then screened to highlight regions of 2CAH that are preferentially hit by the small proteins. It was shown that these preferred regions significantly overlap with biological interfaces (23). A modified PDB file, with the resulting scores generated per residue according to this procedure, was submitted to ENDscript for effective display (Supplementary Figure 1, below). One can notice that the predicted regions of interaction, automatically highlighted in the representation produced by ENDscript, encompass most of the observed intermolecular interfaces. The PyMOL session file corresponding to this demonstrative illustration was produced within 20 seconds of computation time.

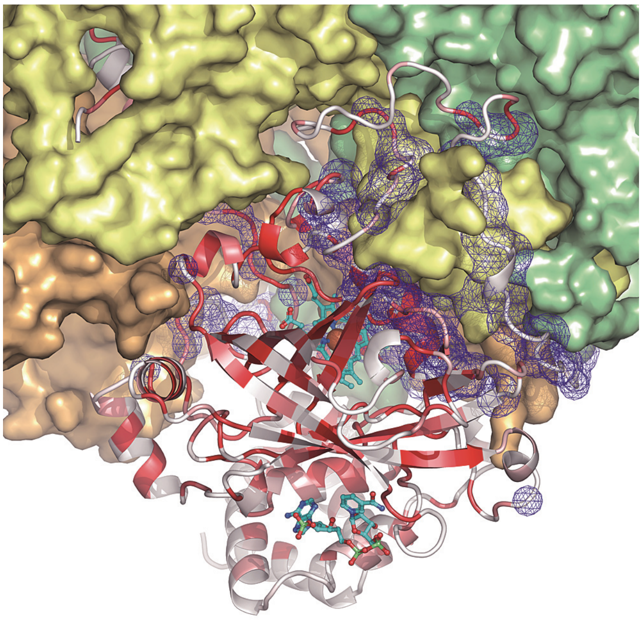

Supplementary Figure 1. Example of ENDscript capabilities to portray a structure-related property imported from an external source.
View of the biological tetrameric assembly of 2CAH. One monomer is presented by a Cartoon representation with the same color ramping as in Figures 1C and 1D. The three other monomers are represented by their solvent accessible surfaces (in salmon, yellow and green). The blue mesh embodies the regions of protein:protein interaction predicted with an arbitrary docking approach.

Supplementary References:

23. Martin, J. and Lavery, R. (2012) Arbitrary protein-protein docking targets biologically relevant interfaces. *BMC biophysics*, 5, 7, doi:10.1186/2046-1682-5-7, PMID:22559010, PMCID:PMC3441232.

24. Ritchie, D.W. and Venkatraman, V. (2010) Ultra-fast FFT protein docking on graphics processors. *Bioinformatics (Oxford, England)*, 26, 2398-2405, doi:10.1093/bioinformatics/btq444, PMID:20685958.
