# Supplementary figures and images for: Deciphering key features in protein structures with the new ENDscript server
Source: Nucleic Acids Res. 2014 Apr 21;42(Web Server issue):W320–4. doi: 10.1093/nar/gku316 (PMC4086106; doi:10.1093/nar/gku316)

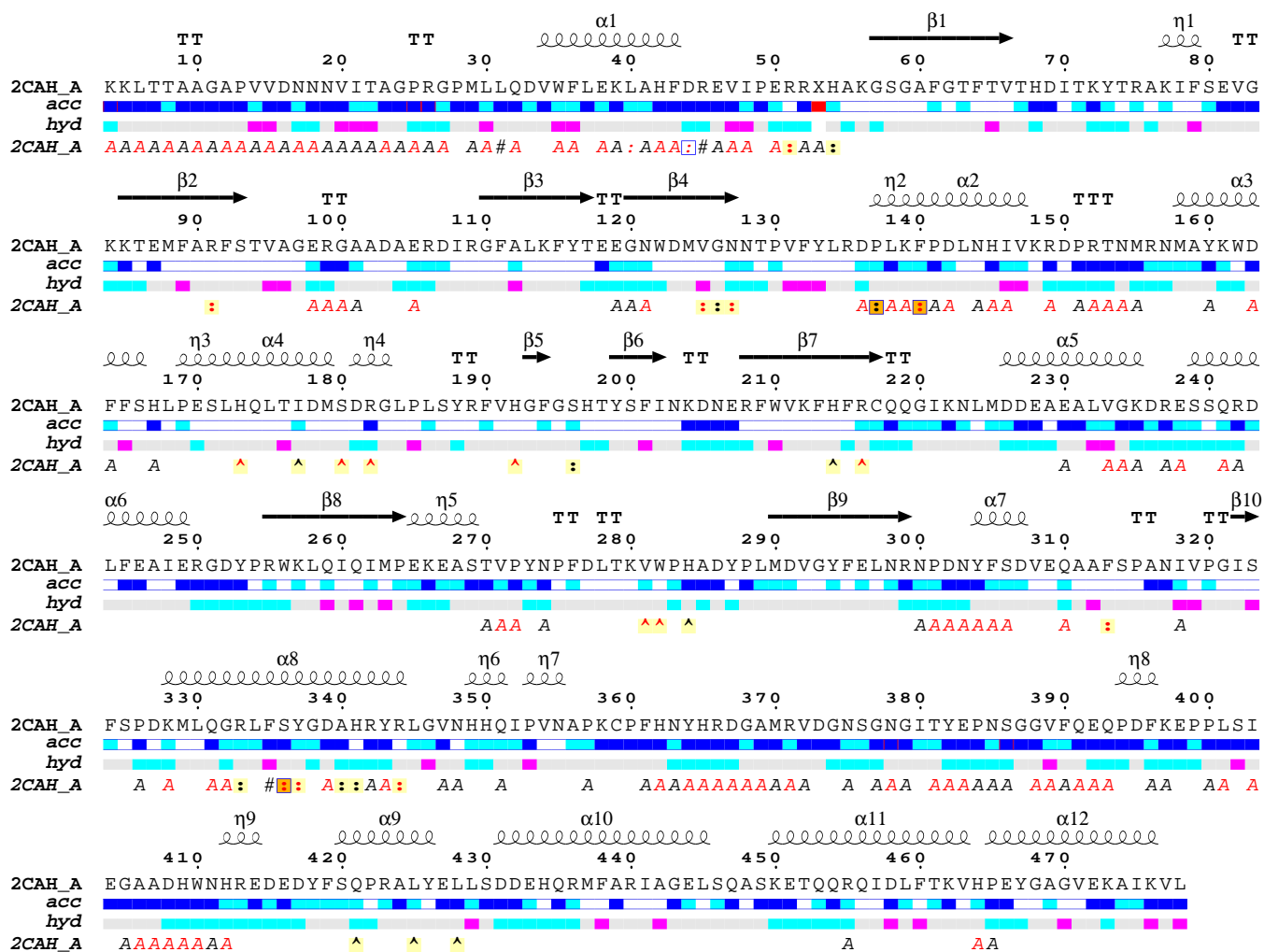

Supplement: Supplementary Data [file supp_gku316_nar-00222-web-b-2014-File005.pdf]

[illegible]

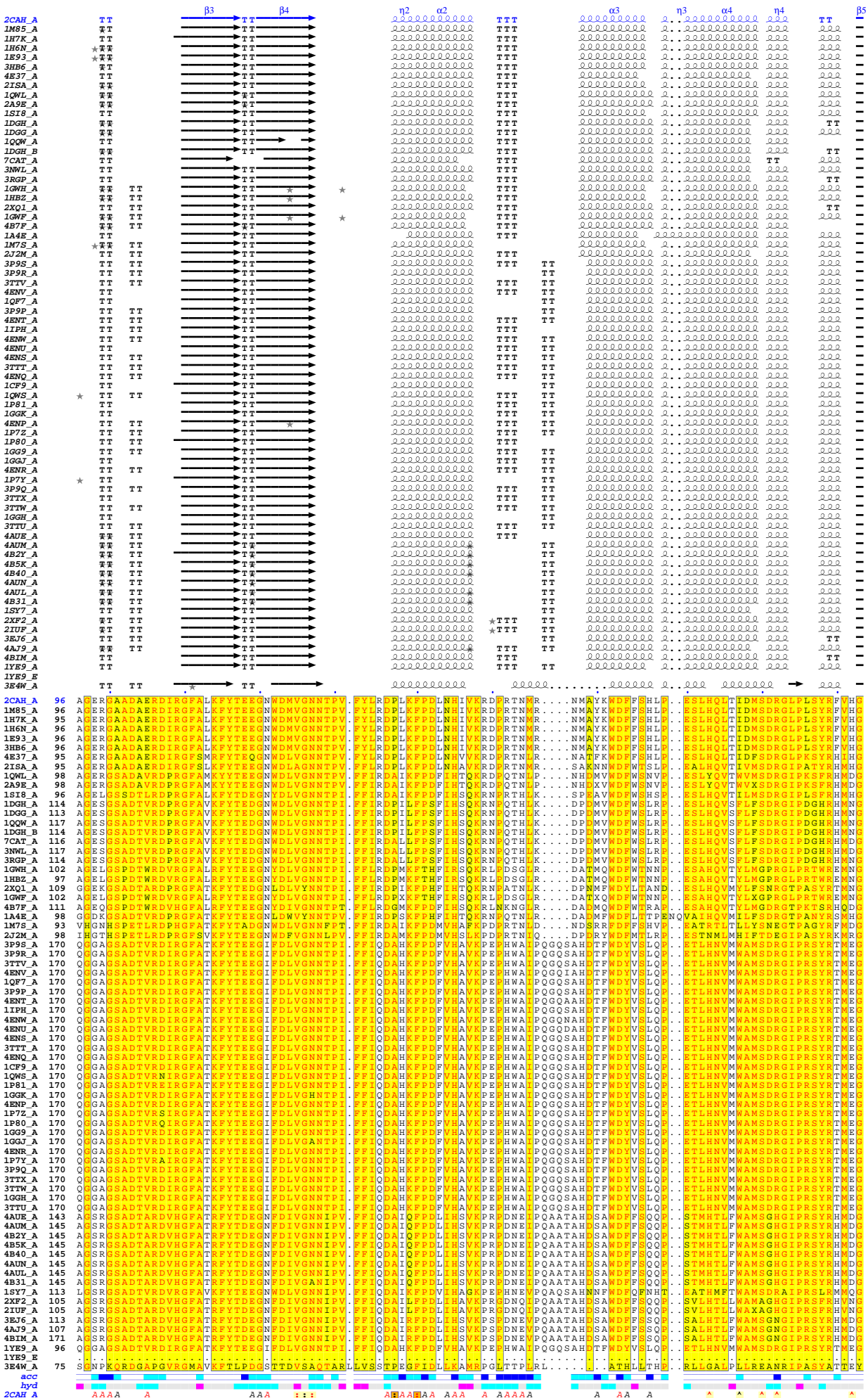

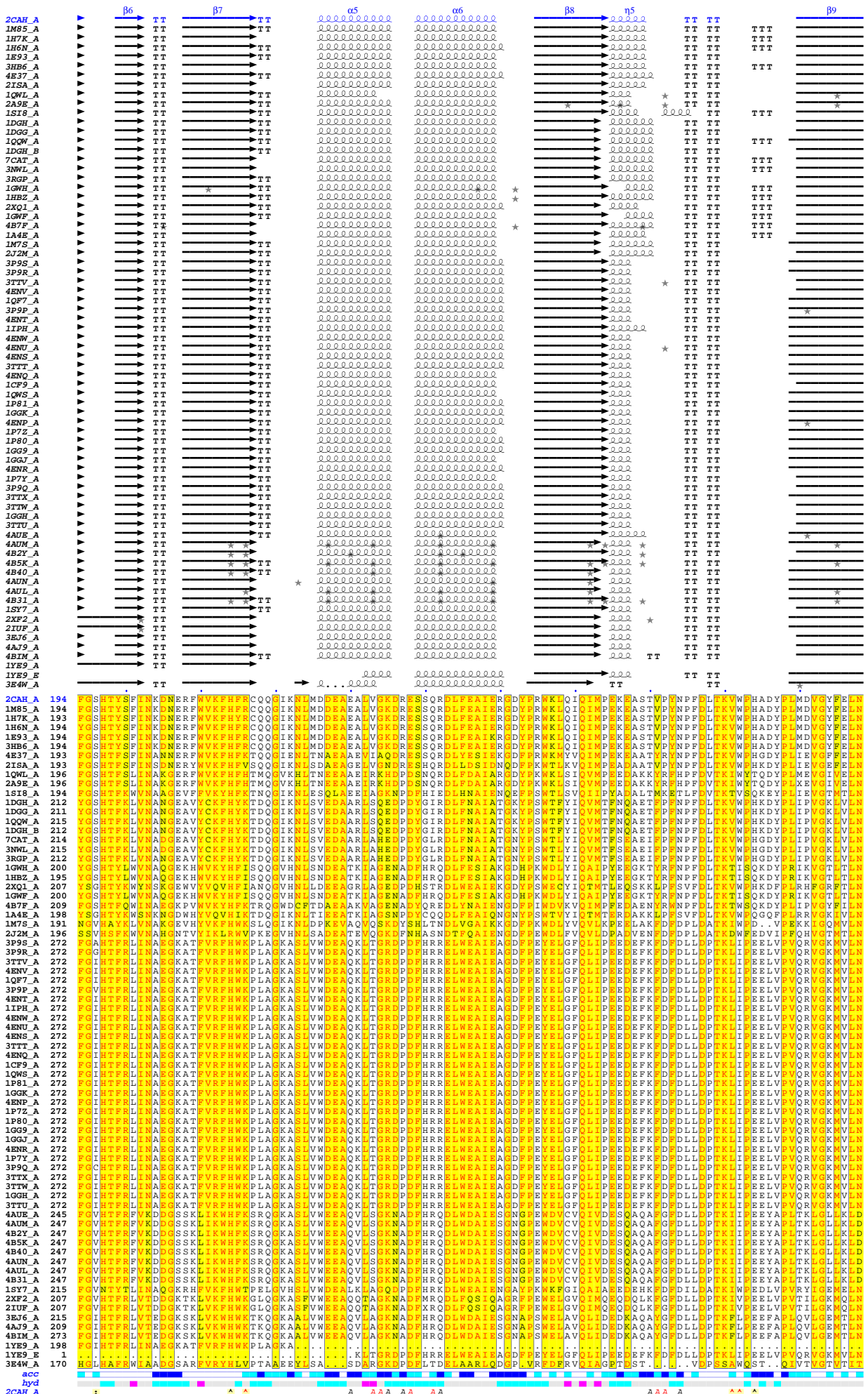



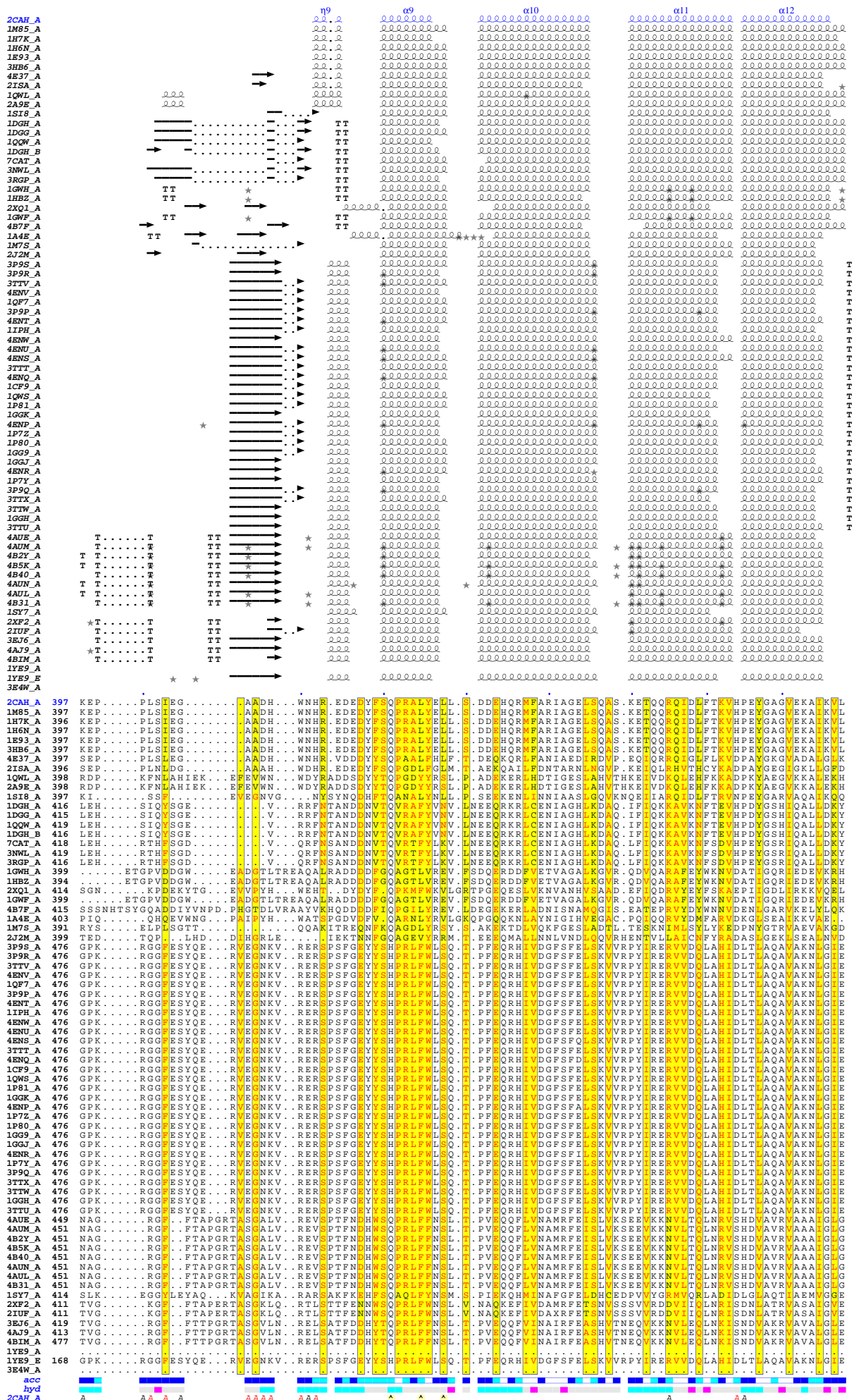

Supplement: Supplementary Data [file supp_gku316_nar-00222-web-b-2014-File006.pdf]
